# Model for Developing Educational Research Productivity: The Medical Education Research Group

**DOI:** 10.5811/westjem.2015.9.27306

**Published:** 2015-11-12

**Authors:** Marcia Perry, Laura Hopson, Joseph B. House, Jonathan P. Fischer, Suzanne Dooley-Hash, Samantha Hauff, Margaret S. Wolff, Cemal Sozener, Michele Nypaver, Joel Moll, Eve D. Losman, Michele Carney, Sally A. Santen

**Affiliations:** * University of Michigan, Department of Emergency Medicine, Ann Arbor, Michigan; † University of Michigan, Department of Emergency Medicine, Children’s Emergency Services, Ann Arbor, Michigan; ‡ University of Michigan, Department of Public Health, Ann Arbor, Michigan

## Abstract

**Introduction:**

Education research and scholarship are essential for promotion of faculty as well as dissemination of new educational practices. Educational faculty frequently spend the majority of their time on administrative and educational commitments and as a result educators often fall behind on scholarship and research. The objective of this educational advance is to promote scholarly productivity as a template for others to follow.

**Methods:**

We formed the Medical Education Research Group (MERG) of education leaders from our emergency medicine residency, fellowship, and clerkship programs, as well as residents with a focus on education. First, we incorporated scholarship into the required activities of our education missions by evaluating the impact of programmatic changes and then submitting the curricula or process as peer-reviewed work. Second, we worked as a team, sharing projects that led to improved motivation, accountability, and work completion. Third, our monthly meetings served as brainstorming sessions for new projects, research skill building, and tracking work completion. Lastly, we incorporated a work-study graduate student to assist with basic but time-consuming tasks of completing manuscripts.

**Results:**

The MERG group has been highly productive, achieving the following scholarship over a three-year period: 102 abstract presentations, 46 journal article publications, 13 MedEd Portal publications, 35 national didactic presentations and five faculty promotions to the next academic level.

**Conclusion:**

An intentional focus on scholarship has led to a collaborative group of educators successfully improving their scholarship through team productivity, which ultimately leads to faculty promotions and dissemination of innovations in education.

## BACKGROUND

Education research and scholarship are essential for promotion of faculty as well as dissemination of new educational practices; however, it presents many challenges. While there are a number of resources for faculty development,^1^ for many faculty scholarship remains a daunting prospect.^2^,^3^ Educational faculty spend the majority of their time on their education mission, leaving little time for scholarly pursuits. As a result, educators may lag on the scholarship essential for academic promotion. To combat this, creative tools are needed to promote scholarly activity for educational faculty.

Research literature on academic productivity indicates that institutional factors may play a larger role in determining research output than individual factors.^4^ This insight has led to educational innovations about how to foster research environments that better promote productivity. Bland et al. found that factors promoting scholarship include (1) clear goals that serve as a coordinating function, (2) a distinctive culture of research emphasis with assertive participation, (3) frequent communication, (4) accessible resources, and (5) leadership with expertise and skill.^5^ This work has served as a guide for research groups since its publication and has contributed to a growing body of education research literature. Questions remain regarding how to effectively implement the principles of effective research environments using these research group guidelines.

## OBJECTIVES

As educational faculty face increasing demands on their time, it is imperative that new and creative models are developed to foster more productive research environments. The objective of this educational advance is to promote faculty scholarly productivity. We describe how the incorporation of research group guidelines to promote successful research through a multi-pronged approach led to scholarly productivity.^5^ This method can provide a valuable template for other departments to follow.

## EDUCATIONAL ADVANCE

The Medical Education Research Group (MERG) consists of faculty leaders from our emergency medicine (EM) residency, fellowship, and clerkship programs. Other non-leadership faculty, EM residents, and pediatric EM fellows with a focus on education also participated. The group was formed in 2008. Initially, the group was led by a successful basic scientist with a challenge to the educational faculty to look for scholarship in their day-to-day work. Skill building and faculty development (invited presentations on education research topics, participation of faculty in Medical Education Scholars Program were the focus of those first few years. She left to become division director elsewhere in 2009. The group’s early work and focus on scholarship was propelled forward in late 2011 with the start of a new associate chair for education who had specific experience in education research and a track record of publishing educational scholarship. Thirteen faculty attend regularly, with about 8–10 present at each meeting. There is no mandate, incentive or tracking of participation.

The key components of each meeting include several areas. During the discussions of new projects, ideas are shared, research plans developed, and teams formed. Group mentoring occurs through the detailed discussions surrounding project development. The research work is implemented by the team outside of MERG, with updates and problems brought back to the group. These are often related to curriculum changes in the educational programs.

For each project there is an intentional process over sequential meetings. One of our projects was the outcome of a residency program change that entailed a switch from confidential to faculty-identified evaluation of residents. The MERG group decided to study the impact of this change, which resulted in the eventual completion of an abstract and manuscript submission. To achieve this kind of goal, we take these steps: 1) We develop the research questions, data to be collected, and determine who will compose the research team. 2) At each meeting we include an update of current projects to ensure continued project momentum and completion. 3) There is intentional scholarship planning surrounding national meetings. Several months before a national meeting submission deadline, we brainstorm and plan for didactic and research submissions including current and new projects. 4) If we identify a knowledge deficit or an educational need of the group, we will read an article or invite a local expert for the purpose of skill development. For example, a local expert on survey design was invited to present on key elements of successful survey research and also review ongoing projects.^6^–^8^

### Clear Goals as a Coordinating Function through the Formation of a MERG

MERG meets monthly with the goal of bringing scholarly inquiry to the usual tasks required by medical educators such as curriculum design and trainee assessment, thereby turning our usual work into scholarship. This pushes the group to consider how the usual work of education can be scholarly. Thus, many changes and innovations in curriculum or educational processes are accompanied by hypothesis generation, data collection and analysis leading to research studies and other scholarship. This creates a distinctive culture of research emphasis and scholarly inquiry so that whenever we consider a change, there is the accompanying question of how we are going to measure the effectiveness. For example, when we began using Accreditation Council for Graduate Medical Education Milestones, several faculty queried how prepared graduating students would be for the new milestones and who was responsible for ensuring preparedness. We saw an opportunity to assess the preparedness and assessment of medical students during the transitions to internship. As a result, two projects were implemented and published.^9^,^10^ Thus, we study the impact of educational innovations implemented in our programs and submit the work for peer-review and dissemination.

### Leadership with Expertise and Skill

Initially senior faculty with expertise in medical education as well as clinical or bench research expertise provided mentorship. As the group has gained experience, peer mentorship is predominant. As individuals develop areas of expertise, they are often tasked to present back to the group to share knowledge. In addition, we have begun to see the initial faculty participants in MERG taking the lead on their own independent projects and actively including junior members who are just making an initial foray into scholarship. The group now includes the educational leadership of all of the education domains from medical students, residents, and fellows, as well as individuals in other domains looking to cross into educational scholarship.

### Team Science

We work as a team. Sharing projects has led to motivation, accountability, and work completion. Our monthly meetings serve as brainstorming sessions for new projects, research skill building, and tracking work completion. The team projects and monthly meetings serve to provide a positive culture with assertive participation and frequent communication as described by Bland. We intentionally include multiple author teams to create a division of labor, such as writing different sections of the manuscript or submitting the accompanying MedEDPortal publications, so that the workload, responsibility, and recognition are distributed among all team members.

### Resources

Often faculty do not have the bandwidth or passion for writing required to bring manuscripts to completion, leading to failure of dissemination of educational scholarship. Therefore, when we realized that we had over 10 published abstracts that had not been turned into manuscripts we tried a new process to address this challenge. We theorized that we should be able to use a graduate student to help us translate our ideas and abstracts into published papers. The group worked with a Master’s of Public Health graduate student for about 25 hours a month for eight months at low cost as it was subsidized by the federal work-study program. (The cost was less than $1,000 for about five hours a week). His role was to help bring the research ideas to completion through performance of the literature review, clarification of the study concept, data interpretation, and drafting of the manuscript. He completed the initial draft that was usually substantially revised by the first or anchor author who then coordinated the revisions and final product. He helped the group maintain a tight timeline to bring to completion one research project per month.

Recognizing that additional resources were needed to augment faculty effort, we undertook additional steps 1) We used undergraduate research assistants for data collection (three projects). As in many academic institutions, there are undergraduate students who can collect data. For example, one of the projects collected patient surveys scoring resident communication. If this resource is not available, then it would be important to steer away from projects that require specific hands-on data collection. 2) At times our residency administrative support was used for retrieving, organizing, or entering data. For example, an administrative assistant might download the faculty scoring of intern milestones for analysis. The amount of work required was variable. 3) It is often the last stages of submission that creates delays. For that purpose, we trained an administrative assistant to do referencing with EndNote, ^TM^ maintain an education-based EndNote library, and perform final proofs of manuscripts. 4) Occasional unpaid statistical support was used from the Department of Medical Education (six projects). 5) As needed, we have used outside resources by inviting outside scholars to speak with the group. For example, we invited Dr. (name blinded) to assist us with standard setting our competency examination and Dr. (name blinded) to clarify the process of MedEdPortal submissions. These resources were accelerators to publication without which the group would have been much slower to publish.

## IMPACT & EFFECTIVENESS

### Impact

To measure the impact of this educational innovation, we collected the following information for the MERG group as a whole and for each faculty member: number of abstracts accepted at regional, national, or international meetings, didactic presentations, papers accepted or published, papers submitted but under review or not yet accepted, grants, and promotions from 2011–2014. We excluded book chapters because they are not peer reviewed. Studying the impact of MERG was determined not to be regulated by the IRB.

Over the past four years, the MERG group has been highly productive. The team effort resulted in 102 presented abstracts, 46 publications, and 35 didactic presentations (Table). We anticipate additional publications as a number of papers are currently under review. In addition, we have encouraged trainee scholarship. Indirect impact through skills development and scholarship was also evident in the fact that members of the group are contributing to four grants totaling over 10 million dollars (grant sources: Simulation Center-internal, Interprofessional Center-internal, the Department of Defense, the American Medical Association).

The intentional focus on medical education research has led our collaborative group of educators to successfully promote our scholarship, which will contribute to faculty promotion. (Five members of our group have been promoted.) In addition, because we are deliberate in our assessment of all our educational innovations we are able to refine our curricula and ultimately create a better learning environment for our trainees. The model, following Bland’s research guideline of creating a group with clear goals that meets monthly to work as a team and adds resources as needed, was found to be both feasible and effective.

Three key attributes of this model lie at the heart of its success. First, this group facilitates valuable mentorship between members that may not have taken place otherwise. Mentorship, both by senior faculty and by peers, is a vital aspect of growth and learning and has allowed group members to develop into more highly-skilled researchers.^11^–^15^ Second, this group facilitates effective teamwork. This teamwork allows new faculty to get involved in research projects more easily, keeps faculty accountable to each other for producing results, and provides a venue for creating, quickly vetting and refining research ideas. Third, this group identifies education gaps within the group and addresses these needs through presentations from visiting scholars, discussions within the group, reference materials and articles. This creates a community of educational practice and level of discussion because members are educated on topics they would not have had the opportunity to learn about otherwise and raises the level of discussion and implementation.

It is our hope that this innovation will inspire other institutions to create new education research groups based on this model. To that aim, a number of key challenges that this group has faced are outlined below. These lessons can provide insights for other institutions into how to create research groups of their own.

A major benefit of this group, as outlined above, is that it facilitates mentoring between members. Due to time constraints on medical students and residents it can be difficult for them to participate. Increased effort needs to be employed to include trainees in projects and meetings. Another challenge was that due to the small and resource-limited nature of this group, demand for administrative support was at times higher than available capacity.

A further challenge is that such a group may require an organizational catalyst or educational expert to drive formation and commitment until the cultural change is established. This leadership may be available through virtual mentorship or use of non-education research experience or educationalists outside the department. Additional difficulties may be faced by smaller and more resource-limited institutions wanting to establish a similar research model. However, we found that this model did not consume significant resources and was effective at promoting scholarly activity. We believe, therefore, that this innovation presents a useful method of increasing academic output in any emergency department that wishes to implement it.

## CONCLUSION

An intentional focus on scholarship has led to our collaborative group of educators successfully increasing their scholarship through team productivity, which ultimately leads to faculty promotions.

## Figures and Tables

**Table:** Summary of impact on academic output after the establishment of a collaborative group of emergency medicine educators.

Scholarship for the 13 MERG faculty	Average per MERG member* (SD)	Total for the group
Abstract presentations (+/−publication)	14 (17)	102
Publications (accepted or published)	4.9 (5.7)	46
Papers in review or revision	3.4 (2.9)	14
MedEd portal publications	2.2 (2.9)	13
MedEdPortal in review		2
Scholarship with students, residents, fellows		60
Didactic sessions	3.5 (4.9)	35
Total scholarship (limitation: this number may double count abstracts and publications)	28.7 (32.1)	212
Faculty members promoted (4 instructor to assistant, 1 associate to full)		5
Grants		4

*MERG,* medical education research group; *SD,* standard deviation

* Average is calculated by total number divided by the number of MERG members (13).
